# Investigation of publication bias in meta-analyses of diagnostic test accuracy: a meta-epidemiological study

**DOI:** 10.1186/1471-2288-14-70

**Published:** 2014-05-23

**Authors:** W Annefloor van Enst, Eleanor Ochodo, Rob JPM Scholten, Lotty Hooft, Mariska M Leeflang

**Affiliations:** 1 Dutch Cochrane Centre and Department of Clinical Epidemiology, Biostatistics and Bioinformatics, Academic Medical Center, Amsterdam, The Netherlands; 2 Department of Clinical Epidemiology, Biostatistics and Bioinformatics, Academic Medical Center, Amsterdam, The Netherlands; 3 Current address: Dutch Cochrane Centre, Julius Center for Health Sciences and Primary Care, University Medical Center Utrecht, Utrecht, The Netherlands

**Keywords:** Publication bias, Diagnostic test accuracy, Funnel plot, Meta-analyses, Small study-effects

## Abstract

**Background:**

The validity of a meta-analysis can be understood better in light of the possible impact of publication bias. The majority of the methods to investigate publication bias in terms of small study-effects are developed for meta-analyses of intervention studies, leaving authors of diagnostic test accuracy (DTA) systematic reviews with limited guidance. The aim of this study was to evaluate if and how publication bias was assessed in meta-analyses of DTA, and to compare the results of various statistical methods used to assess publication bias.

**Methods:**

A systematic search was initiated to identify DTA reviews with a meta-analysis published between September 2011 and January 2012. We extracted all information about publication bias from the reviews and the two-by-two tables. Existing statistical methods for the detection of publication bias were applied on data from the included studies.

**Results:**

Out of 1,335 references, 114 reviews could be included. Publication bias was explicitly mentioned in 75 reviews (65.8%) and 47 of these had performed statistical methods to investigate publication bias in terms of small study-effects: 6 by drawing funnel plots, 16 by statistical testing and 25 by applying both methods. The applied tests were Egger’s test (n = 18), Deeks’ test (n = 12), Begg’s test (n = 5), both the Egger and Begg tests (n = 4), and other tests (n = 2). Our own comparison of the results of Begg’s, Egger’s and Deeks’ test for 92 meta-analyses indicated that up to 34% of the results did not correspond with one another.

**Conclusions:**

The majority of DTA review authors mention or investigate publication bias. They mainly use suboptimal methods like the Begg and Egger tests that are not developed for DTA meta-analyses. Our comparison of the Begg, Egger and Deeks tests indicated that these tests do give different results and thus are not interchangeable. Deeks’ test is recommended for DTA meta-analyses and should be preferred.

## Background

When the decision to publish the results of a study depends on the nature and direction of the results, publication bias arises. There are many forms and reasons for publication bias such as time-lag bias (due to delayed publication), duplicate or multiple publications, outcome reporting bias (selective reporting of positive outcomes) and language bias [1-6]. These forms of biases tend to have more effect on small studies and contribute to the phenomenon of “small study-effects” [7]. This means that published studies with small sample sizes tend to have larger and more favourable effects compared to studies with larger sample sizes. This is a threat to the validity of a systematic review and its meta-analyses [8].

For intervention reviews graphical and statistical methods have been developed to investigate if the results of the meta-analyses of the review might be affected by publication bias in terms of small study-effects. A well-known graphical method is the funnel plot examination [9]. This method aims to construct a scatter plot of the study effect sizes on the horizontal axis against some measure of each study’s size or precision on the vertical axis. The dots in this plot together look like an inverted funnel. An asymmetric funnel is an indication for publication bias. Since the plot gives a visual relationship between the effect and study size, its interpretation is subjective. This is not an issue when statistical tests are used to detect funnel plot asymmetry. There are eight tests available [10], but the test of Begg [11], and the test of Egger [12] are probably most common. They have been cited more than 2,500 (Begg) and 7,300 times (Egger) [13]. The test of Begg assesses if there is a significant correlation between the ranks of the effect estimates and the ranks of their variances. The test of Egger uses linear regression to assess the relation between the standardized effect estimates and the standard error (SE). For both tests a significant result is an indication that the results might be affected by publication bias. These and other methods have been developed especially for systematic reviews of intervention studies and are not automatically suitable for reviews of diagnostic test accuracy (DTA) studies [9].

DTA meta-analyses have different characteristics making assessment of the potential for publication bias more complicated than for intervention reviews. The diagnostic odds ratio (DOR) usually takes high values, while intervention effects are usually quite small. Secondly, the SE of the DOR depends on the proportion of positive tests, but this proportion is influenced by the variation in threshold amongst different studies. Thirdly, the number of diseased and non-diseased patients are usually unequally divided, which reduces the precision of a test accuracy estimate while in RCTs equal numbers of participants are allocated to an intervention or control group. Investigating whether meta-analyses of DTA studies have been influenced by publication bias in terms of small study-effects is challenging [14]. Even diagnostic meta-analyses free of publication bias might have an asymmetric funnel plot due to other reasons like the threshold effect. In addition, bivariate meta-analysis is recommended for DTA meta-analyses [13] but bivariate methods for the detection of publication bias are currently not available. Hence, the DOR is used as an univariate alternative to detect publication bias, but not for the final meta-analysis that assesses the accuracy.

Knowledge of the mechanisms that may induce publication bias in diagnostic studies or empirical evidence for the existence of publication bias is scarce. Selective publication of accuracy studies based on the magnitude of the sensitivity or specificity doesn’t seem to be very plausible. In addition, what parameter is most important (and thus driving possible selective publication) depends also on the place of the test in the clinical pathway and it’s role [15]. Korevaar et al. compared prospective registered diagnostic studies to the publications. They concluded that failure to publish and selective publication were prevalent in diagnostic accuracy studies but the dataset was too small to draw firm conclusions [16]. Brazelli and colleagues, however, tracked a cohort of conference abstracts and did not find evidence of publication bias in the process that occurs after abstract acceptance [17].

In 2002, Song and colleagues proposed that tests developed for intervention reviews, like Begg’s and Egger’s methods could also be used to detect publication bias in DTA reviews. They suggested to use the natural logarithm of the DOR (lnDOR) and plot it against its variance or SE and test for asymmetry [18]. In 2005, however, Deeks and colleagues conducted a simulation study of tests for publication bias in DTA reviews. They concluded that existing tests that use the SE of the lnDOR can be seriously misleading and often have false positive results [19]. The Cochrane Handbook for Systematic Reviews of Diagnostic Test Accuracy explicitly mentions not to use methods like the Begg or Egger tests and argues that it is best to use the test proposed by Deeks [14]. This test has been developed especially for test accuracy reviews and proposes plotting the lnDOR against 1/effective sample size (ESS)^1/2^ and testing for asymmetry of this plot. The ESS is a function of the number of diseased (n_1_) and non-diseased (n_2_) participants: (4n_1*_n_2_)/(n_1_ + n_2_). The ESS takes into account the fact that unequal numbers of diseased and non-diseased reduce the precision of the test accuracy estimates [19]. Using the ESS instead of total sample size will reduce the unequal numbers of diseased and non-diseased and thereby enhance the precision of the accuracy estimates. The Cochrane Handbook, however, points out that even Deeks’ test has low power to detect small study-effects when there is heterogeneity in the DOR. As heterogeneity in DTA reviews is the rule rather than the exception the Cochrane Handbook warns the authors against misinterpretation of this test [14].

Because little is known about the mechanisms behind and the existence of publication bias in DTA studies it is difficult for reviewers to select the correct method for addressing selective publication. In addition, the interpretation of the results of the various methods and incorporating those results in the formulation of the conclusions of the review is even more challenging. Different tests to identify publication bias in terms of small study-effects are expected to report different results. However, since all tests aim at assessing the same concept, publication bias, the differences should be minimal. A simulation study did show that differences in test outcomes are, however, quite substantial [19]. This has not been confirmed in empirical data. To understand more about the assessment of publication bias in DTA reviews led us to following objectives.

The primary objective of this study was to assess which existing tests for publication bias have been used and to what extent the results of these tests have been incorporated in the review. A second objective was to compare the results of existing methods for the detection of publication bias in non-simulated data to assess if these various methods would provide similar results.

## Methods

### Study selection

MEDLINE was searched through the interface of PubMed for DTA reviews published between September 2011 and January 2012. The search was performed in February 2012 by one author (EO) using a search filter for systematic reviews available from PubMed combined with a methodological filter for DTA studies: (systematic[sb] AND (("diagnostic test accuracy" OR DTA[tiab] OR "SENSITIVITY AND SPECIFICITY"[MH] OR SPECIFICIT*[TW] OR "FALSE NEGATIVE"[TW] OR ACCURACY[TW]))) [20].

### Eligibility criteria

Articles were eligible for inclusion if they systematically assessed the diagnostic accuracy of a test or biomarker and were published in English. Methods to investigate publication bias are developed to investigate publication bias in meta-analyses [14]. Therefore, the selection was further limited to reviews that included a meta-analysis. Availability of the two-by-two tables of the included studies was not amongst the inclusion criteria to generate a representative cohort of reviews without possible selection on high level of reporting and perhaps review quality [21]. Studies that assessed the accuracy by means of individual patient data were excluded as the methodology of such studies differs from those of meta-analyses on a study level.

### Definitions of assessment of publication bias

In determining if authors would assess publication bias in their reviews, we scored if authors described a method how they would investigate publication bias like drawing a funnel plot or performing a test for publication bias. If the methods were lacking but the results of a publication bias assessment were described, it was also scored as an investigation of publication bias. We regarded the results of the assessments as being incorporated in the discussion of the reviews when the authors described how publication bias might have affected the results of their reviews.

### Data extraction

An online standardized data extraction form was used to extract data. We first piloted the form among all team members. After everyone agreed on the data-extraction form, the actual extraction was then done by one reviewer (WE). An online randomization program selected a random sample of one third of the reviews that was checked by a second reviewer (ML, FW, RS). In case the number of differences between reviewers was <3%, no further data checking was done. Disagreements were resolved by discussion.

For the first objective, data was extracted on all reported matters concerning assessing publication bias: if the authors had planned to assess or assessed publication bias and the described methods, the number of studies that were included in the test, results of the test, and consideration of the test results with the interpretation of the pooled results. When authors had no intention to test for publication bias, the review was screened to find a reason for this and if the possible threat of publication bias was discussed or considered to formulate the conclusion. For the second objective, the two-by-two tables (true positives, false positives, false negatives, true negatives) were extracted when reported in the reviews or when they could be derived from other results (e.g. number of diseased and non-diseased combined with the sensitivity or specificity).

### Comparison of tests for publication bias

The secondary objective of this study was to assess the concordance of publication bias test results in empirical data. We applied three univariate tests: the Begg test and Egger test because these are cited frequently, and Deeks’ test because this test has been developed for DTA meta-analyses and is currently recommended in the Cochrane DTA Handbook [14]. The tests were performed as follows:

•Begg’s test: rank correlation of the lnDOR with the variance of the lnDOR [11];

•Egger’s test: linear regression of lnDOR with the standard error of the lnDOR weighted by the inverse variance of the lnDOR [12];

•Deeks’ test: linear regression of lnDOR with 1/ESS^1/2^ weighted by the ESS [19].

Concordance between the results of tests defined as both having or not having a significant result (p-value <0.05) was presented as Cohen’s weighted kappa, taking into account agreement due to chance. The simulation study of Deeks et al. indicated that tests would more frequently perform differently when the pooled DOR is 38 or higher [19]. In addition tests need sufficient power to perform optimal which may be relevant for concordance. Therefore, we performed logistic regression to study whether concordance between tests was related to a pooled DOR >38, the number of primary studies, or the number of included patients. Analyses were performed in the statistical program R [22].

## Results

**Figure 1:**
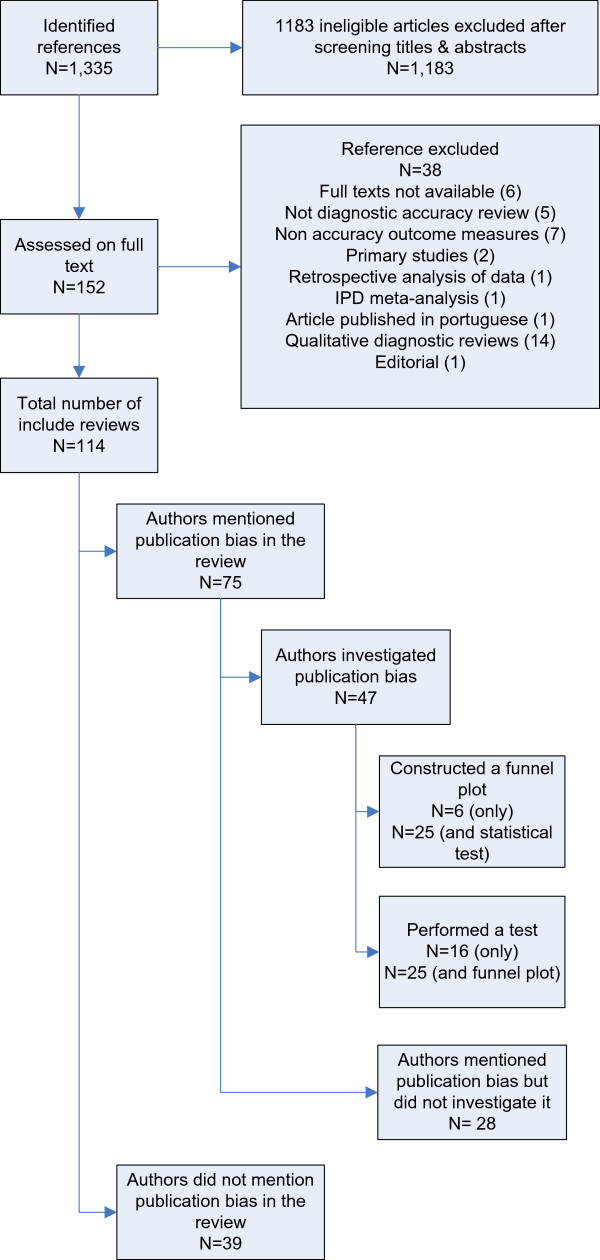
Flow chart of the selection process and characters of the included studies.

We identified 1,335 references of potential eligible studies, of which 152 were assessed on full text for eligibility. Finally, 114 DTA reviews were included for the current study. Details of the selection process are presented in Figure 1. There was optimal agreement (98.6%) when the second reviewer checked the data.

Publication bias was explicitly mentioned in 75 reviews (65.8%). Of these, 47 (62.7%) had performed methods to investigate publication bias in terms of small study-effects: 6 by investigating funnel plots, 16 by statistical testing for asymmetry and 25 by applying both methods. Table 1 gives details on how publication bias was investigated per review.

**Table 1:** Overview of the applied methods to investigate publication bias

**Reference**	**Funnel plot**		**Results of the funnel plot**	**Test**	**Results of the test**	**Remarks**
	***x-axis***	***y-axis***				
Chang 2011 [23]	-	-	-	Egger	3/7	
Chang 2012 [24]	Sensitivity Specificity	SE	Not considered	Begg Egger	1/2 1/2	
Cheng 2012 [25]	lnDOR	1/root(ESS)	No publication bias	Not specified	0/2	
Descatha 2012 [26]	lnDOR	1/root(ESS)	No publication bias	Deeks	0/2	
Dong 2011 [27]	-	-	-	Begg Egger	0/1 0/1	Results for a second diagnostic tool were not presented.
Dym 2011 [28]	Sensitivity Specificity	1/SE	Inconclusive 2/2	-	-	
Gao 2011 [29]	lnDOR	SE(lnDOR)	1/2	Begg	1/2	
Gargiulo 2011 [30]	lnDOR	1/root(ESS)	Not considered	Deeks	1/2	
Glasgow 2012 [31]	lnDOR	1/Var(lnDOR)	0/2	-	-	
Gong 2011 [32]	Sensitivity	Sample size	Inconclusive 2/2	-	-	Plots had too low power.
Hernaez 2011 [33]	-	-	-	Deeks	0/1	
Inaba 2012 [34]	lnDOR RR^1^	SE(lnDOR) SE(RR)	1/2	Egger^2^	1/2	Level of significance p-value <0.10
Kobayashi 2012 [35]	DOR	SE(DOR)	2/2	Begg	0/2	Both plots indicated publication though the tests were not significant.
Li 2011 [36]	-	-	-	Egger	1/1	Publication bias was detected for a subgroup by the test.
Li 2012 [37]	-	-	-	Egger	1/1	
Lu 2011 [38]	lnDOR	1/root(ESS)	Not considered	Deeks	0/1	
Lundstrom 2011 [39]	-	-	-	Egger	0/1	
Luo 2011 [40]	lnDOR	1/root (ESS)	Not considered	Egger	0/3	
Manea 2012 [41]	-	-	?	Begg	?	Results were not presented
Mao 2012 [42]	-	-	-	Egger	1/1	
Marton 2012 [43]	Not specified	Not specified	Not considered	Egger	1	One plot and test to investigate two diagnostic tools
Mathews 2011 [44]	AUC(ROC)^3^	SE(AUC(ROC))	0/2	Egger	0/2	
McInnes 2011 [45]	lnDOR	SE(lnDOR)	-	Egger	0/1	
Meader 2011 [46]	-	-	-	Egger	?	Results were not presented.
Mitchell 2011 [47]	-	-	-	Begg	?	Results were not presented.
Onishi 2012 [48]	-	-	-	Egger	2/2	
Papathanasiou 2012 [49]	lnDOR	SE(lnDOR)	Not considered	Begg	1/1	
Plana 2012 [50]	lnDOR	1/root(ESS)	Not considered	Deeks	0/2	Not identified by tests Plots was not used to draw conclusions.
Qu 2011 [51]	logDOR	Sample size	?/2	-	-	Results of funnel plots were inconclusive, too low power.
Sadeghi 2012 [52]	logDetectionRate^4^ logSensitivity	SE(logDetect Rate) SE(logSens)	0/2	Egger	0/2	
Sadigh 2011 [53]	-	-	-	Deeks	0/1	
Summah 2011 [54]	lnDOR	SE(lnDOR)	1/1	Egger	1/1	
Sun 2011 [55]	-	-	-	Deeks	0/1	No publication bias was detected by the test.
Takakuwa 2011 [56]	lnDOR	1/root (ESS)	1/1	Deeks	0/1	Identified by plot though not by test.
Thosani 2012 [57]	lnDOR	SE(lnDOR)	Not considered	Egger	2/2	Plots were not used to draw conclusion.
Tomasson 2012 [58]	Difference in arcsine^5^	Precision(Dif. in arcsine)	2/2	Egger	0/2	Identified by plots though not by tests.
Trallero-Araguas 2012 [59]	-	-	-	Deeks	0/1	
Wang 2011 [60]	-	-	-	Begg Egger	0/2 0/2	
Wang 2012 [61]	lnDOR	SE(lnDOR)	7/7	Egger	3/7	
Wang 2012 [62]	lnDOR	SE(lnDOR)	0/2	Begg Egger	0/2	
Wang 2012 [63]	lnDOR	SE(lnDOR)	0/2	-	-	
Wu 2012 [64]	lnDOR	1/root(ESS)	0/1	Deeks	0/1	
Xu 2011 [65]	-	-	-	Egger	0/1	
Xu 2011 [66]	lnDOR Standardized effect^6^	SE(lnDOR) Precision(St. effect)	0/2	Begg-Mazumdar Harbord-Egger	0/2	
Ying 2011[67]	lnDOR	1/root(ESS)	0/2	Deeks	0/2	
Yu 2012 [68]	lnDOR	SE(lnDOR)	1/1	-	-	
*Zhang 2011*[69]	lnDOR	1/root(ESS)	0/1	Deeks	0/1	

^1^RR = Relative Risk; It is unclear which estimates were used to calculate the RR.

^2^The methods section specifies that the Egger test has been used though the text of the figures specified the Begg test.

^3^AUC(ROC) = Area Under the Curve (AUC) of the Receiving Operating Characteristic (ROC).

^4^There was no definition for Detection Rate specified in the article.

^5^Difference in arcsine = Transformed ratios of arcsine for those with rise in Anti-Neutrophil Cytoplasmic Antibody (ANCA) and persistent ANCA among subjects who had relapse and those who did not.

^6^Standardized effect was explained as differentiating benign and malignant lymph nodes.

In 28 reviews (24.6%), publication bias was mentioned though it was not investigated. Fifteen of these reviews (13.2%) mentioned why they did not investigate publication bias. These reasons were: because the methods to investigate publication are lacking and can provide misleading results (n = 7), lack of power to detect publication bias (n = 6), too heterogeneous results to further investigate publication bias (n = 1), and underlying principles of publication bias in DTA studies are not yet known and publication bias can therefore not be investigated (n = 1).

### Funnel plots

In the 31 reviews that presented funnel plots, different concepts were plotted. Funnel plots were constructed per test under review (n = 20), per target condition (n = 2) (e.g. MRI to detect colon cancer or to detect lung cancer) and for different accuracy measures of a test (n = 5) (e.g. sensitivity and specificity). In four reviews the authors made comparisons of the accuracy of several clinical tests but used one single plot to investigate publication bias (two of these, however, did construct different funnel plots for different accuracy measures).

The axes that were used to plot were diverse. On the horizontal axis the DOR (DOR or lnDOR) was most often used (n = 24), but also other accuracy parameters like sensitivity or ROC area (n = 5). Four reviews used other parameters (relative risk, detection rate, difference in the arcsine between two groups, and standardized effect). On the vertical axis we found a variety of precision measures: SE(lnDOR) (n = 12), 1/variance(lnDOR) (n = 1), 1/(ESS)^1/2^ (n = 10), and sample size (n = 2). For two reviews the authors had constructed two plots per test: one plot with the sensitivity on the horizontal axis with 1/SE(sens) on the vertical axis and one plot of the specificity on the horizontal axis with 1/SE(spec) on the vertical axis.

### Statistical tests

In 41 reviews a statistical test was performed to investigate publication bias. The applied tests were Egger’s test (n = 18), Deeks’ test (n = 12), Begg’s test (n = 5), both the Egger and Begg test (n = 4), and both the Begg-Mazumdar and Harbord’s test [70]. One review did not specify which test was used. Two reviews used the trim and fill method to adjust for small study-effects. The median number of studies in the analyses was 13 (IQR 9–19) with a range from 4 to 118. Two review authors mentioned that a minimum of twenty homogeneous studies was required to perform a test [71,72].

Authors that had applied the Egger test most often reported significant results indicating the existence of publication bias (37.2%), while authors that applied the Deeks test least reported significant results in identifying publication bias (6.7%) (Table 2).

**Table 2:** Reported results of different tests to assess small study in the included reviews (n=41)

**Type of test**	**Small study effects**		
	***Identified (%)***	***Not identified (%)***	**Total**
**Begg**	3 (18.8)	13 (81.2)	16
**Egger**	16 (37.2)	27 (62.8)	43
**Deeks**	1 (6.7)	14 (93.3)	15
**Begg-Mazumdar**	0	1 (100)	1
**Harbord-Egger**	0	1 (100)	1
**All tests**	20 (26.0)	56 (74.0)	76

In 8 reviews the authors used more than one test to examine publication bias. The results of both tests in these reviews were in agreement with one another, though the p-values could be quite diverse (e.g. investigation of publication bias of FDG-PET studies to detect in breast cancer: Begg’s p = 0.462, Egger’s p = 0.052 [63] or imaging studies to detect osteomyelitis: Begg’s p = 0.392 and Egger’s p = 0.063 [60]).

### Incorporation of results in the discussion

The results of investigation of publication bias were discussed in 25 out of 47 reviews that assessed publication bias. Six reviews based their conclusion about publication bias only on the plots, as they had not performed a test. One of these reviews concluded the existence of publication bias, two concluded no existence of publication and three were inconclusive about the influence of publication bias for their review. In reviews that had constructed a funnel plot and performed a test, the conclusions were based on the combination (funnel plot and test) or only on the test. In cases of disagreement between the results of a funnel plot and a test, all authors emphasized on the test results.

In fourteen reviews, the issue of publication bias was raised as a limitation to the results while five reviews concluded that there was no risk of publication bias. Two reviews discussed that the assessment had increased their confidence in the results of their review, though four reviews mentioned that it had affected the results and that these results should be considered cautiously.

Eleven reviews that did not assess publication bias mentioned that the possible existence of publication bias could be a limitation to the results of their review. In these reviews, authors stated that comprehensive searching, placing no limits on study quality or language could be used as precautions to prevent effects of publication bias. Two reviews also mentioned that excluding conference proceedings could have introduced publication bias.

### Comparison of tests to detect publication bias

**Figure 2:**
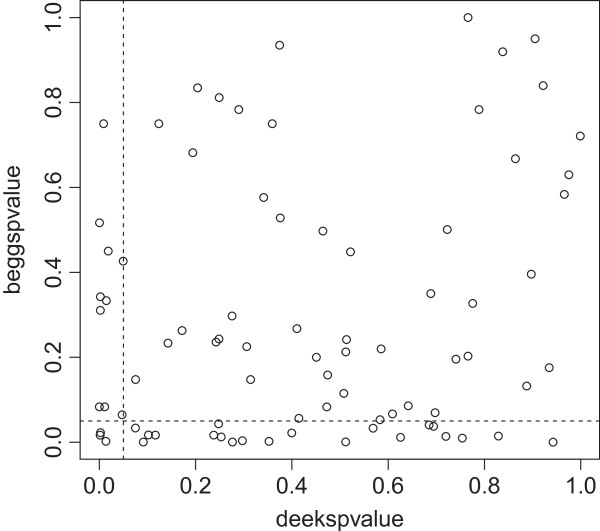
**Comparison of the p-values of the Begg test (y-axis) and Deeks’ test (x-axis) in 92 meta-analyses.** The dotted lines indicate a p-value of 0.05. Concordance between tests was 67% (κ = −0.039; 95% CI −0.23 to 0.15).

**Figure 3:**
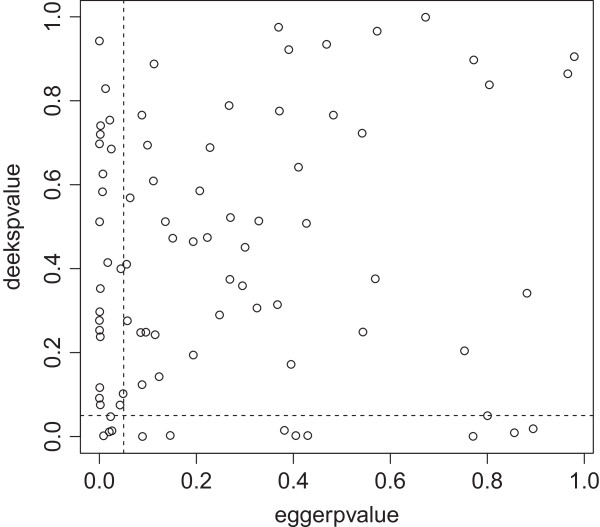
**Comparison of the p-values of the Egger test (y-axis) and Deeks’ test (x-axis) in 92 meta-analyses.** The dotted lines indicate a p-value of 0.05. Concordance between tests was 66% (κ = −0.002; 95% CI −0.2 to 0.19).

**Figure 4:**
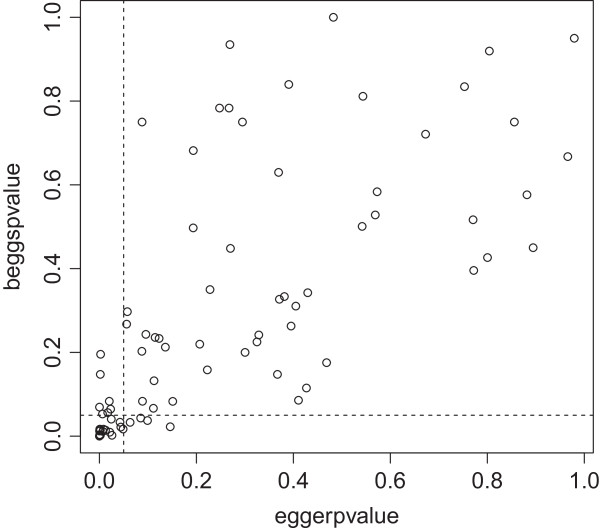
**Comparison of the p-values of the Begg test (y-axis) and the Egger test (x-axis) in 92 meta-analyses.** The dotted lines indicate a p-value of 0.05. Concordance between tests was 87% between tests (κ = 0.68; 95% CI 0.51 to 0.86).

We were able to obtain two by two tables of 52 reviews, including 92 different meta-analyses. There was moderate concordance between the various tests for publication bias in terms of the presence or absence of significance (Figures 2, 3 and 4). Concordance of the Begg and Egger tests was significantly better depending on the number of included studies (OR 1.09; 95% CI 1.03 to 1.10). The number of included participants or a DOR >38 did not have a significant association with the concordance of tests (Table 3).

**Table 3:** Odd ratio’s for the association between several factors and the concordance between tests

***Factor***	***Begg – Deeks OR (95% CI)***	***Egger –Deeks OR (95% CI)***	***Begg – Egger OR (95% CI)***
Number of participants	1.00 (0.99 to 1.00)	1.00 (1.00 to 1.00)	1.00 (1.00 to 1.00)
Number of studies	0.96 (0.98 to 1.02)	1.00 (0.99 to 1.01)	1.09 (1.03 to 1.10)*
DOR > 38	1.02 (0.93 to 1.15)	0.955 (0.85 to 1.20)	0.999 (0.96 to 1.00)

*P-value <0.001.

## Discussion

Most authors of DTA reviews (65.8%) are concerned about publication bias. In 41.2% of the included reviews methods were applied to investigate publication bias. Funnel plots were constructed with a diversity of parameters on the axes and were sparsely used in isolation to formulate conclusions about the existence of publication bias. Forty-one reviews assessed publication bias with a statistical test. The Deeks test that is especially developed for reviews of diagnostic accuracy was only used in 12 reviews (10.5%). In 18 reviews (15.8%), the results of the publication bias assessment led to less confidence in the results. Our replication of three tests to detect publication bias (Begg, Egger and Deeks) using empirical data indicated that the results of the tests frequently conflict with one another. The study of Deeks et al. showed that a type 1 error is likely to occur in both the Begg and the Egger tests when the threshold for test positivity, the disease prevalence or the magnitude of the accuracy estimates varies between the included studies, especially when the DOR is high (DOR > 38), which is present in almost every DTA review [19]. Although, we cannot be sure in which reviews the test results were accurate and in which they were false, it seems likely that these two tests may have led to an overestimation of the presence of publication bias.

The number of reviews investigating publication bias seems to have increased over time. In 2002, Song and colleagues investigated how authors assessed publication bias in a sample of 20 reviews including 28 DTA meta-analyses. They concluded that none of the included reviews had investigated publication bias and that only 4 out of 20 reviews had considered its likelihood in the discussion [18]. Furthermore, in 2011, Parekh-Bhurke et al. conducted a review to examine the approaches that are used to deal with publication bias in different types of systematic reviews published in 2006. They reported that only 26% of all reviews used statistical methods to assess publication bias [73]. Of the 50 diagnostic reviews that were included in this study, nine (18%) used funnel plot asymmetry to investigate publications bias and in three (6%) a statistical test. These numbers are remarkably lower than found in our study. This could be the result of the increased awareness of the possible threat of publication bias in DTA reviews.

The increased awareness of publication bias is a positive development, but the drawback here is that the majority of review authors use tests that are not fit for DTA meta-analyses. Our evaluation of 92 meta-analyses indicated that both the Begg and Egger tests give more significant results than Deeks’ test. This result is in line with the expectation based on the simulation study by Deeks et al. [19]. The trim and fill method was used in two reviews only. This method removes the most extreme small studies on the side of the desired outcome direction in the funnel plot, and recomputes the effect size at each iteration until the plot is symmetrical [17]. A recent simulation study in DTA meta-analyses showed that the trim and fill method is more powerful than other tests like the Begg, Egger or Deeks test to detect possible publication bias [74]. Therefore, this method may be used more frequently in future.

Our study is limited by the fact that we based our results on what is reported in the publications. It is possible that funnel plots were constructed for more reviews but were not included in the publication. This may have led to an underestimation of the actual number of reviews that constructed a funnel plot. Secondly, our own assessment of publication bias in the meta-analyses is based on the data reported in the reviews but it is, of course, not clear if any of the meta-analyses were actually biased by publication bias as a gold standard is currently absent [14].

As correctly mentioned in some of the reviews included in our study, little is known about the actual existence of selective publication of DTA studies [75]. There is no evidence regarding the existence of biases like language bias or time lag bias in the DTA setting, nor if these biases affect the accuracy measures in the same way as they affect the effect of interventions. It could be argued that depending on the purpose of the test either the sensitivity or the specificity are more affected by selective publication than the DOR, and tests for publication bias should perhaps be directed to these two accuracy parameters. A special situation of selective publication may occur with non-inferiority designs for diagnostic test accuracy. This study design aims to compare the diagnostic accuracy of a new diagnostic test with a standard test and is based on the difference in paired partial area under the ROC curve. This difference can be tested with Bayesian methods that result in a p-value [76,77]. Because of this p-value, this design may be more susceptible to non-publishing negative findings and as such induces publication bias. However, as long as the mechanisms behind publication bias of diagnostic studies are not well understood, it is understandable that some reviewers decided not to formally investigate how publication bias may have affected their meta-analysis.

Prospective registration of intervention studies has been shown to be an effective measure to reduce selective publication or at least make it more transparent to investigators. At the moment, prospective registration is advocated for diagnostic accuracy studies but not a prerequisite like it is for intervention studies in order to be considered for publication in journals associated with the International Committee of Medical Journal Editors (ICMJE) [78]. Empirical studies to assess and understand the mechanisms that may induce publication bias in DTA studies, however, are needed. A cohort of prospective diagnostic studies could be followed and the dissemination of study results may be compared to the study characteristics and results. Optimization could be achieved if prospective registration of diagnostic accuracy studies would be mandatory. This may not be beneficial for all types of diagnostic studies. For example diagnostic data are often collected as part of daily clinical care and retrospectively analysed. Still, prospective registration of at least the prospective diagnostic studies could improve the understanding of the process of selective publication of DTA studies and identify underlying mechanisms. This knowledge is needed for valid interpretation of results of meta-analyses of diagnostic studies.

## Conclusions

We found that most DTA reviewers struggle how to deal with publication bias in their reviews. Suboptimal tests like Egger’s and Begg’s are frequently used, while the interpretation of the test results are rarely linked to the pooled results. Deeks’ tests should be preferred to assess publication bias in DTA meta-analyses and interpretation of a significant test result should be done within the perspective that we are unaware whether publication bias exists for DTA studies. We advise authors of DTA reviews to try to avoid the introduction of publication bias and apply thorough methods for identifying primary studies, alongside regular searches in electronic biomedical databases. This entails identifying grey literature, contacting experts and searching for conference proceedings. Prospective registration of diagnostic studies with a prospective design could be helpful in the perspective of selective reporting.

## Abbreviations

ANCA: Anti-Neutrophil Cytoplasmic Antibody; AUC: Area Under the Curve; DOR: Diagnostic odds ratio; DTA: Diagnostic test accuracy; ESS: Effective sample size; ICMJE: International Committee of Medical Journal Editors; lnDOR: Natural logarithm of the odds ratio; RR: Relative risk; ROC: Receiving Operating Characteristicl; SE: Standard error; Sens: Sensitivity; Spec: Specificity.

## Competing interests

This research project has not been funded. We have no competing interest to report that could have affected the results of our study.

## Authors’ contributions

WE has contributed to the protocol of the study, data extraction, data-analysis and wrote manuscript. EO contributed to the protocol and performed the search and selection process of studies. She helped with data-checking and contributed to the manuscript. LH has contributed to the protocol of the study and contributed to the manuscript. RS has contributed to the protocol of the study, helped with data-checking and contributed to the manuscript. ML has contributed to the protocol of the study, performed the selection process of studies, helped with data-checking and contributed to the manuscript. All authors read and approved the final manuscript.

## Authors’ information

ML, RS and LH are all involved in the Cochrane DTA working group. Further, the authors declare that they have no competing interests.

## Pre-publication history

The pre-publication history for this paper can be accessed here:

http://www.biomedcentral.com/1471-2288/14/70/prepub
